# CDK5 Regulatory Subunit-Associated Protein 1-like 1 Negatively Regulates Adipocyte Differentiation through Activation of Wnt Signaling Pathway

**DOI:** 10.1038/s41598-017-06469-5

**Published:** 2017-08-04

**Authors:** Kazumi Take, Hironori Waki, Wei Sun, Takahito Wada, Jing Yu, Masahiro Nakamura, Tomohisa Aoyama, Toshimasa Yamauchi, Takashi Kadowaki

**Affiliations:** 1 Department of Diabetes and Metabolic Diseases, Graduate School of Medicine, The University of Tokyo, 7-3-1 Hongo, Bunkyo-ku, Tokyo 113-8655 Japan; 2 Functional Regulation of Adipocytes, Graduate School of Medicine, The University of Tokyo, 7-3-1 Hongo, Bunkyo-ku, Tokyo 113-8655 Japan; 3 Department of Molecular Sciences on Diabetes, Graduate School of Medicine, The University of Tokyo, 7-3-1 Hongo, Bunkyo-ku, Tokyo 113-8655 Japan

## Abstract

CDK5 Regulatory Subunit-Associated Protein 1-like 1 (CDKAL1) was identified as a susceptibility gene for type 2 diabetes and body mass index in genome-wide association studies. Although it was reported that CDKAL1 is a methylthiotransferase essential for tRNA^Lys^(UUU) and faithful translation of proinsulin generated in pancreatic β cells, the role of CDKAL1 in adipocytes has not been understood well. In this study, we found that CDKAL1 is expressed in adipose tissue and its expression is increased during differentiation. Stable overexpression of CDKAL1, however, inhibited adipocyte differentiation of 3T3-L1 cells, whereas knockdown of CDKAL1 promoted differentiation. CDKAL1 increased protein levels of β-catenin and its active unphosphorylated form in the nucleus, thereby promoting Wnt target gene expression, suggesting that CDKAL1 activated the Wnt/β-catenin pathway—a well-characterized inhibitory regulator of adipocyte differentiation. Mutant experiments show that conserved cysteine residues of Fe-S clusters of CDKAL1 are essential for its anti-adipogenic action. Our results identify CDKAL1 as novel negative regulator of adipocyte differentiation and provide insights into the link between CDKAL1 and metabolic diseases such as type 2 diabetes and obesity.

## Introduction

Type 2 diabetes (T2DM) and obesity are major risk factor for cardiovascular disease and caused by genetic and environmental factors. A series of genome-wide association studies have shown that single-nucleotide polymorphisms (SNPs) in the CDKAL1 gene locus are significantly associated with type 2 diabetes^1–4^. In addition, other studies have shown that this locus has an association with body mass index in adults and children^5–8^ or body weight at birth^9, 10^. Wei *et al*. recently reported that CDKAL1 a methylthiotransferase that synthesizes 2-methylthio-*N* ^*6*^-threonylcarbamoyladenosine in tRNA^Lys^(UUU)^11^. By using pancreatic-β-cell-specific knockout mice, the researchers demonstrated that CDKAL1 is essential for accurate translation of the codons for Lys in proinsulin in the β cells, and that its deficiency resulted in aberrant proinsulin synthesis, increased ER stress, decreased insulin secretion and impaired glucose metabolism^11^. Ohara-Imaizumi *et al*. demonstrated by using isolated pancreatic islets from CDKAL1 knockout mice that CDKAL1 controls first-phase insulin exocytosis in β cells by facilitating ATP generation, K(ATP) channel responsiveness and the subsequent activity of Ca^2+^ channels^12^. Okamura *et al*. reported mixed phenotypes of CDKAL1 knockout mice. Findings in the early phase of high-fat feeding include temporal decline in food intake and body weight and protection from insulin resistance. After 20 weeks of high fat feeding, they observed impaired insulin secretion and glucose tolerance, increased insulin resistance, as well as increase of lipid content in the liver and muscle^13^. However, whether CDKAL1 plays any functional role in adipocyte differentiation had not been directly addressed.

In our study, we investigated a role of CDKAL1 in 3T3-L1 adipocyte differentiation. Our present report demonstrates that CDKAL1 is expressed in adipocytes and that CDKAL1 negatively regulates adipocyte differentiation by activating the Wnt signaling pathway.

## Results

### Expression levels of CDKAL1 in adipose tissue and the adipogenic cell line

**Figure 1:**
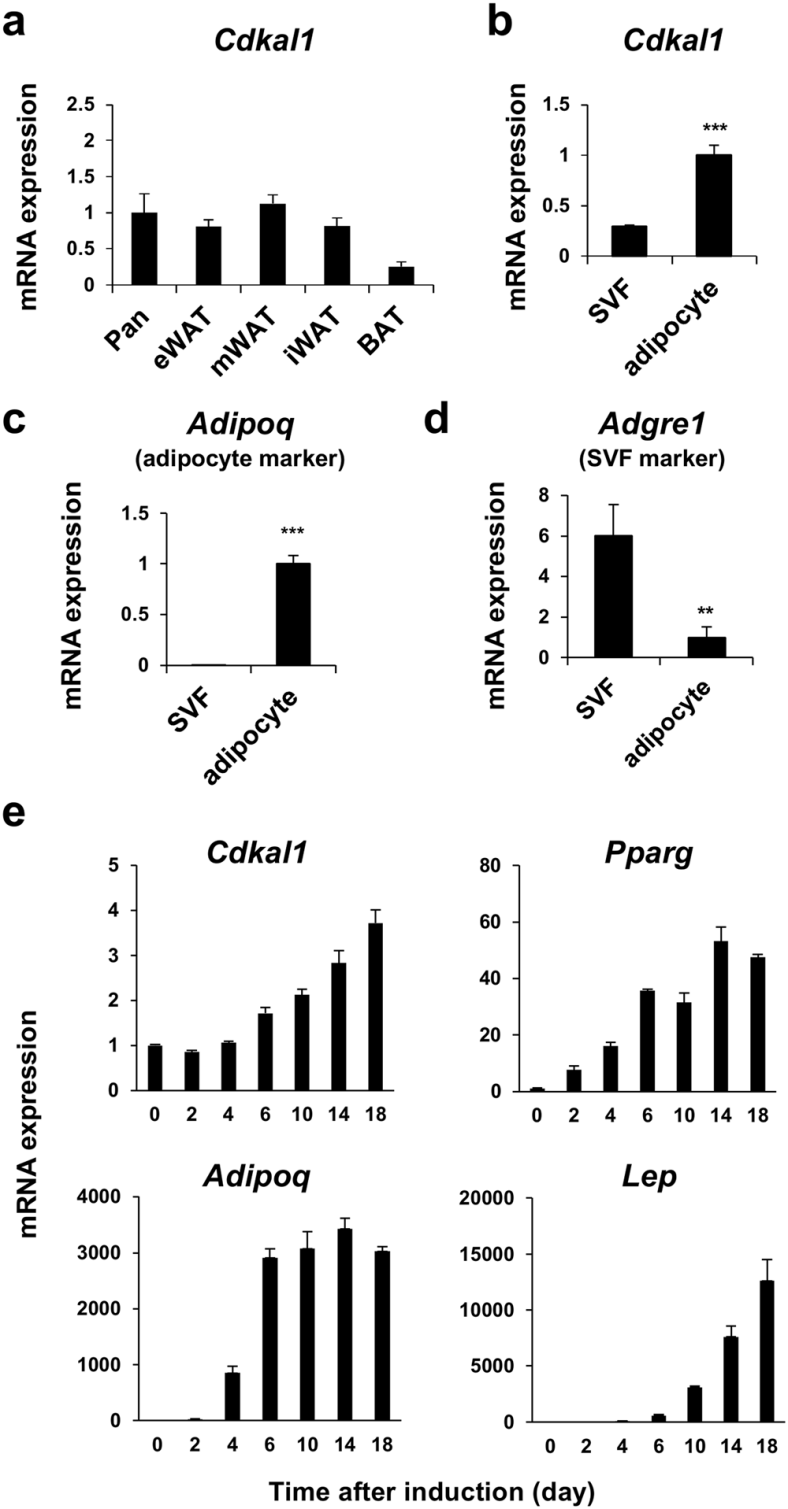
Expression levels of CDKAL1 in adipocytes. (**a**) Expression levels of Cdkal1 in tissues of C57BL/6 J mice. eWAT, epididymal white adipose tissue (WAT); mWAT, mesenchymal WAT; iWAT, inguinal WAT; BAT, brown adipose tissue; Panc, pancreas (n = 4 [BAT], 7 [Pancreas] or 8 [(others]). Expression of Cdkal1 (**b**), Adipoq (**c**) and Adgre1 (**d**) in the stromal vascular fraction (SVF) and adipocytes fraction isolated from eWAT of C57BL/6 J mice (n = 4, **p < 0.01, ***p < 0.001 vs SVF group by Student’s t-test). (**e**) Gene expression levels during adipocyte differentiation of 3T3-L1 cells: upper left, Cdkal1; upper right, Pparg; lower left, Adipoq; lower right, Lep (n = 3).

We first examined expression levels of CDKAL1 in mouse tissues and found that CDKAL1 was expressed in various adipose tissues as abundantly as in the pancreas (Fig. 1a). A fractionation experiment of epididymal adipose tissue showed that CDKAL1 was more expressed in adipocytes than in the stromal vascular fraction (Fig. 1b–d). Consistent with higher expression in mature adipocytes, CDKAL1 levels were increased during adipocyte differentiation of 3T3-L1 cells (Fig. 1e). The induction of CDKAL1 during adipocyte differentiation was less than four-fold and milder than that of PPARγ—the master regulator of adipocyte differentiation—and the marker genes such as adiponectin and leptin (Fig. 1e).

### CDKAL1 suppresses adipocyte differentiation of 3T3-L1 cells

**Figure 2:**
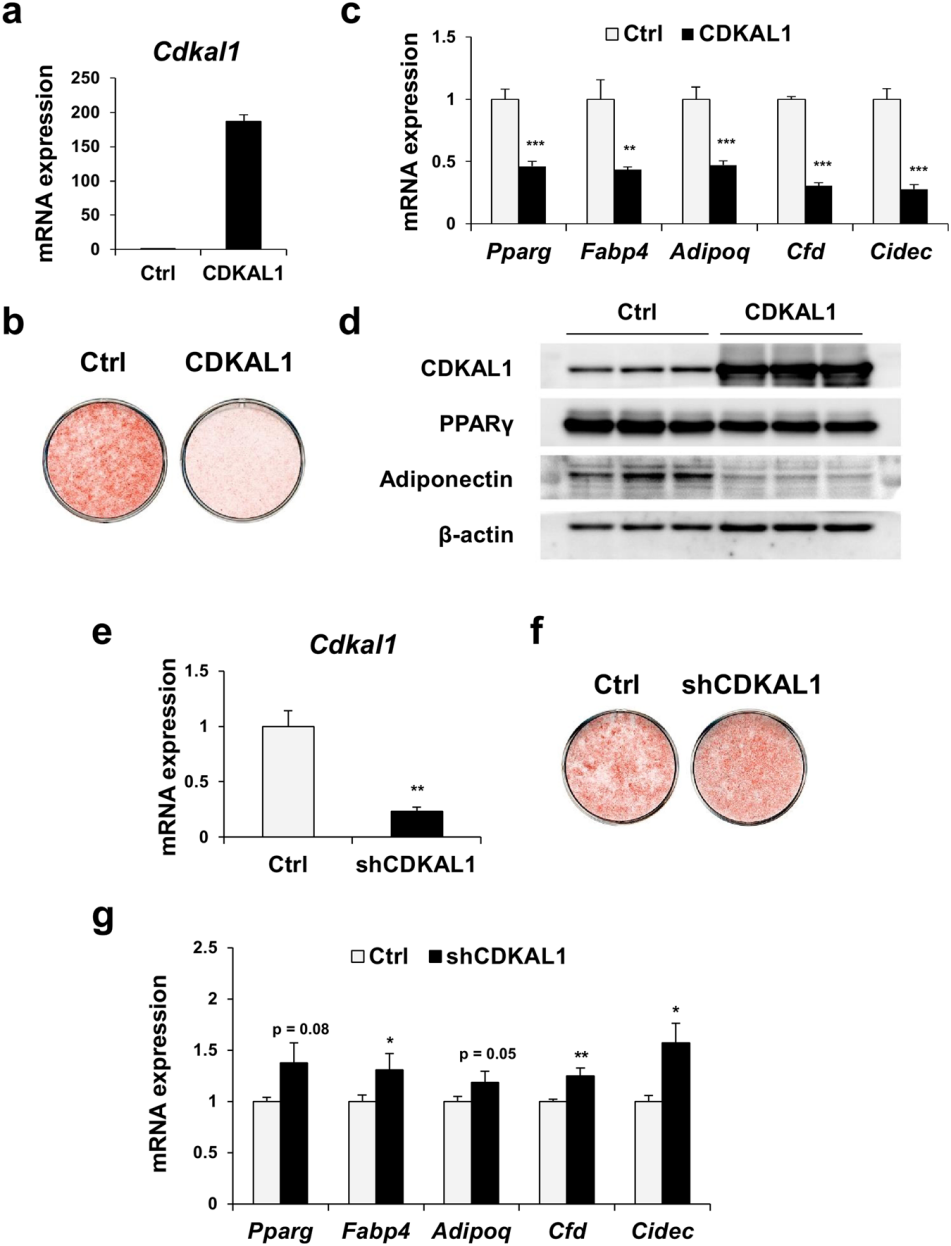
CDKAL1 suppresses adipocyte differentiation. (**a**–**d**) Overexpression of CDKAL1 in 3T3-L1 adipocytes. (**a**) Expression levels of Cdkal1 mRNA levels. (**b**) Plate view of Oil Red O staining (day 7). (**c**) Expression levels of adipocyte specific genes (n = 3, *p < 0.05, **p < 0.01, ***p < 0.001 vs control group by Student’s t-test). (**d**) Western blotting of CDKAL1, PPARγ, adiponectin and β-actin. (**e**–**g**) Knockdown of CDKAL1 by using short hairpin RNA (shRNA) targeting CDKAL1 (shCDKAL1) in 3T3-L1 adipocytes. (**e**) Expression levels of Cdkal1 mRNA levels. (**f**) Plate view of Oil Red O staining. (**g**) Expression levels of adipocyte specific genes (n = 3, *p < 0.05, **p < 0.01 vs control group by Student’s t-test).

To directly test the functional role of CDKAL1 in adipocyte differentiation, we made a 3T3-L1 cell line stably overexpressing CDKAL1 by means of retroviral infection and differentiated them into adipocytes (Fig. 2a). Staining with Oil Red O showed that overexpression of CDKAL1 markedly reduced lipid accumulation in differentiated 3T3-L1 (Fig. 2b). Consistent with the change in lipid accumulation, 3T3-L1 cells expressing CDKAL1 showed significant reduction of expression levels of the adipogenic master regulator PPARγ and its target genes including *Fabp4*, *Adipoq* (adiponectin), *Cfd* (adipsin) and *Cidec* (Fig. 2c). Furthermore, western blot analysis showed reduced protein levels of PPARγ and adiponectin in cells overexpressing CDKAL1 (Fig. 2d). In contrast, knockdown of CDKAL1 by short hairpin RNA (shRNA) resulted in increased lipid accumulation and increased expression of the adipogenic genes in 3T3-L1 cells (Fig. 2e–g). We also used shRNA for the luciferase gene as another negative control, and we observed the similar effect of CDKAL1 knockdown on the expression of these genes (Suppl. Fig. 1). These data indicate that CDKAL1 serves as a negative regulator of adipocyte differentiation despite the increase of expression during adipocyte differentiation.

### Suppression of PPARγ expression by CDKAL1 contributes to the anti-adipogenic action of CDKAL1

**Figure 3:**
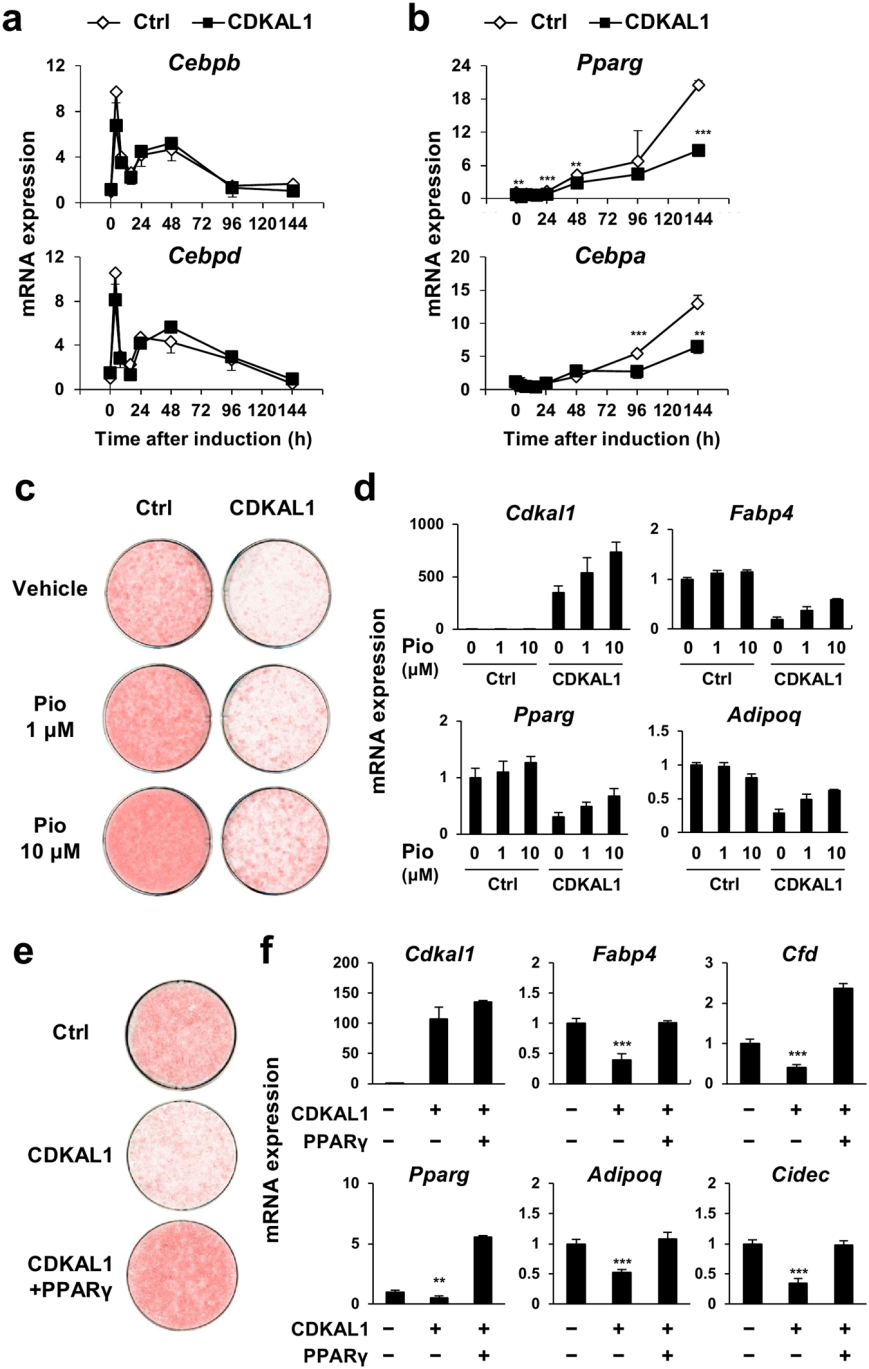
Suppression of PPARγ expression by CDKAL1 contributes to the anti-adipogenic action of CDKAL1. (**a**,**b**) Time course of gene expression levels of early adipogenic regulators, C/EBPβ and C/EBPδ (**a**) and late regulators, PPARγ and C/EBPα (**b**) during adipocyte differentiation of 3T3-L1 cells expressing CDKAL1 (n = 3). (**c**,**d**) Effect of PPARγ agonist pioglitazone on lipid accumulation (**c**) and gene expression (**d**) in differentiated 3T3-L1 cells expressing CDKAL1 (n = 3). (**e**,**f**) Effect of coexpression of PPARγ on lipid accumulation (**e**) and gene expression (**f**) in differentiated 3T3-L1 cells expressing CDKAL1 (n = 3, **p < 0.005, ***p < 0.0005 vs control group by Dunnett’s test).

We next investigated the time course of the induction of key adipogenic transcription factors during adipocyte differentiation. In the control cells, rapid and transient induction of C/EBPβ and C/EBPδ occurs after stimulation with the adipogenic cocktail (Fig. 3a), followed by gradual increase of PPARγ and C/EBPα expression in the late phase of differentiation (Fig. 3b). CDKAL1 significantly blunted the induction of PPARγ and C/EBPα while it did not alter expression levels of C/EBPβ and C/EBPδ (Fig. 3a and b). Pharmacological stimulation of PPARγ with pioglitazone at full dose was not sufficient to reverse the suppression of adipocyte differentiation by CDKAL1 (Fig. 3c and d). In contrast, coexpression of PPARγ with CDKAL1 was able to restore expression of the adipogenic genes and lipid accumulation (Fig. 3e and f). These data suggest that the suppression of PPARγ expression by CDKAL1, at least in part, contributes to the anti-adipogenic action of CDKAL1.

### Activation of Wnt/β–catenin signaling pathway by CDKAL1

**Figure 4:**
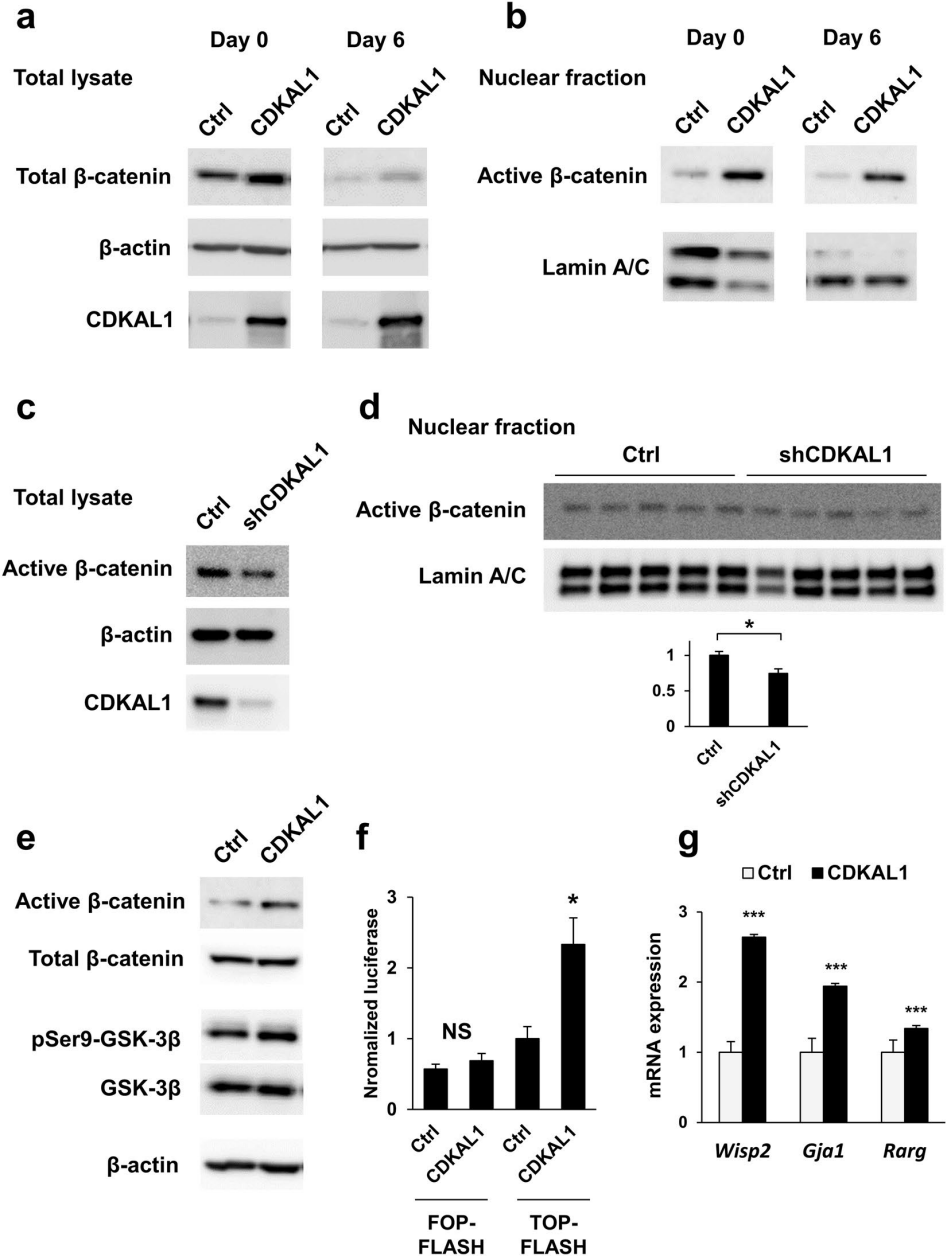
Activation of Wnt/β -catenin signaling pathway by CDKAL1. (**a**) Western blotting of β-catenin in 3T3-L1 expressing CDKAL1 on day 0 and day 6 of differentiation. β-actin was used as an internal control for protein loading. The experiments were repeated multiple times and representative blot was shown. (**b**) Western blotting of active (unphosphorylated) β-catenin in the nuclear fraction in 3T3-L1 expressing CDKAL1. (**c**,**d**) The effect of stable knockdown of CDKAL1 by shRNA in 3T3-L1 cells on protein levels of active β-catenin in total lysate (**c**) and in the nuclear fraction (**d**). The inset graph in (**d**) shows quantification of the active β-catenin (*p < 0.05 vs control group by Student’s t-test). (**e**) Western blotting of β-catenin and inactive form of GSK-3β (phosphorylated at Ser 9) in 3T3-L1 expressing CDKAL1. (**f**) Measurement of Wnt signaling activities by TOPFLASH reporter assay in 3T3-L1 cells. FOPFLASH reporter was used as the negative control (n = 6, *p < 0.05 vs control group by Student’s t-test). (**g**) Gene expression changes of Wnt target genes in 3T3-L1 cells expressing CDKAL1 (12 hours after the initiation of differentiation, n = 3, ***p < 0.001 vs control group by Student’s t-test).

**Figure 5:**
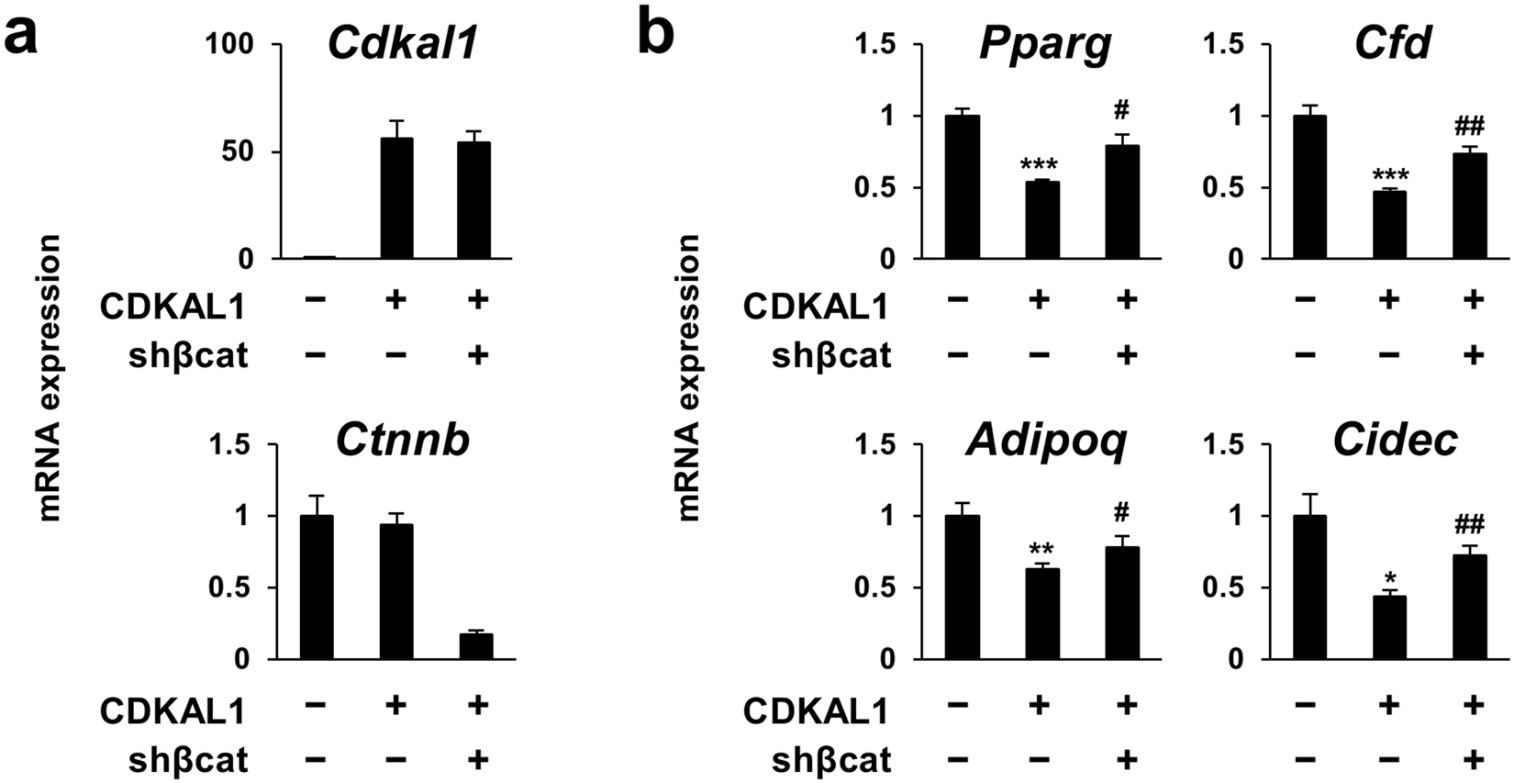
Knockdown of β-catenin compromised the suppression of expression of adipogenic genes by CDKAL1. Gene expression levels of (**a**) CDKAL1 and β-catenin (*Ctnnb*) and (**b**) adipogenic marker genes in 3T3-L1 adipocytes coexpressing CDKAL1 and shRNA targeting β-catenin (n = 3, *p < 0.05, **p < 0.01, ***p < 0.001 vs control group by Student’s t-test; ^#^p < 0.05, ^##^p < 0.01 vs CDKAL1 group by Student’s t-test).

The Wnt/β-catenin signaling pathway is one of the well-characterized inhibitory pathways of PPARγ expression and adipocyte differentiation. As previously documented, β-catenin protein levels decreases during adipocyte differentiation^14^. We found that there was increase of β-catenin in CDKAL1-overexpressing cells during adipocyte differentiation (Fig. 4a). The increase was more prominent when we examine accumulation of unphosphorylated β-catenin (its active form) in the nucleus during adipocyte differentiation (Fig. 4b). Note, the increase of total β-catenin and the active form of β-catenin in the nucleus in CDKAL1-overexpressing cells occurred even before initiation of differentiation by stimulation with the adipogenic cocktail, indicating that the increase of β-catenin by CDKAL1 may play a primary role in the regulation of differentiation, rather than it occurred as a consequence of inhibition of differentiation (Fig. 4a and b, day 0). To test the endogenous role of CDKAL1 on Wnt, we also performed knockdown experiments and we found that stable knockdown of CDKAL1 by shRNA resulted in reduced levels of active β-catenin in ﻿total lysate and in the nucleus (Fig. 4c and d). Similar effects of either overexpression or knockdown of CDKAL1 on active β-catenin were also observed in other adipocyte cell lines such as 3T3-F442A and brown adipocytes (Suppl. Fig. 2). GSK-3β promotes phosphorylation and degradation of β-catenin, and it functions as a negative regulator of the Wnt/β-catenin signaling pathway. We also observed that CDKAL1 overexpression resulted in increased levels of phosphorylated GSK-3β at Ser 9—the inactive form of GSK-3β—suggesting that CDKAL1 increases β-catenin protein levels by suppressing GSK-3β (Fig. 4e). TOPFLASH is a luciferase reporter driven by TCF/LEF response element for monitoring the activity of Wnt/β-catenin signaling. Consistent with the activation of β-catenin, overexpression of CDKAL1 caused significant increase of TOPFLASH reporter activity, but not of the negative control FOPFLASH reporter in 3T3-L1 cells (Fig. 4f). Finally, we performed gene expression analyses and found that overexpression of CDKAL1 resulted in enhanced expression of *Wisp2*, *Gja1* and *Rarg*—target genes of the Wnt signaling pathway in mesenchymal cells^15^ (Fig. 4g). To directly demonstrate that β-catenin is required for CDKAL1’s action, we performed stable knockdown of β-catenin by shRNA. The results showed that the reduction of β-catenin (*Ctnnb*) expression (Fig. 5a) led to reversal of the suppression of PPARγ and its target genes by CDKAL1 overexpression (Fig. 5b). These data collectively suggest that CDKAL1 suppresses adipocyte differentiation by activating the Wnt/β-catenin signaling pathway.

### Conserved cysteine residues in the amino terminal region of CDKAL1 are essential for its anti-adipogenic action

**Figure 6:**
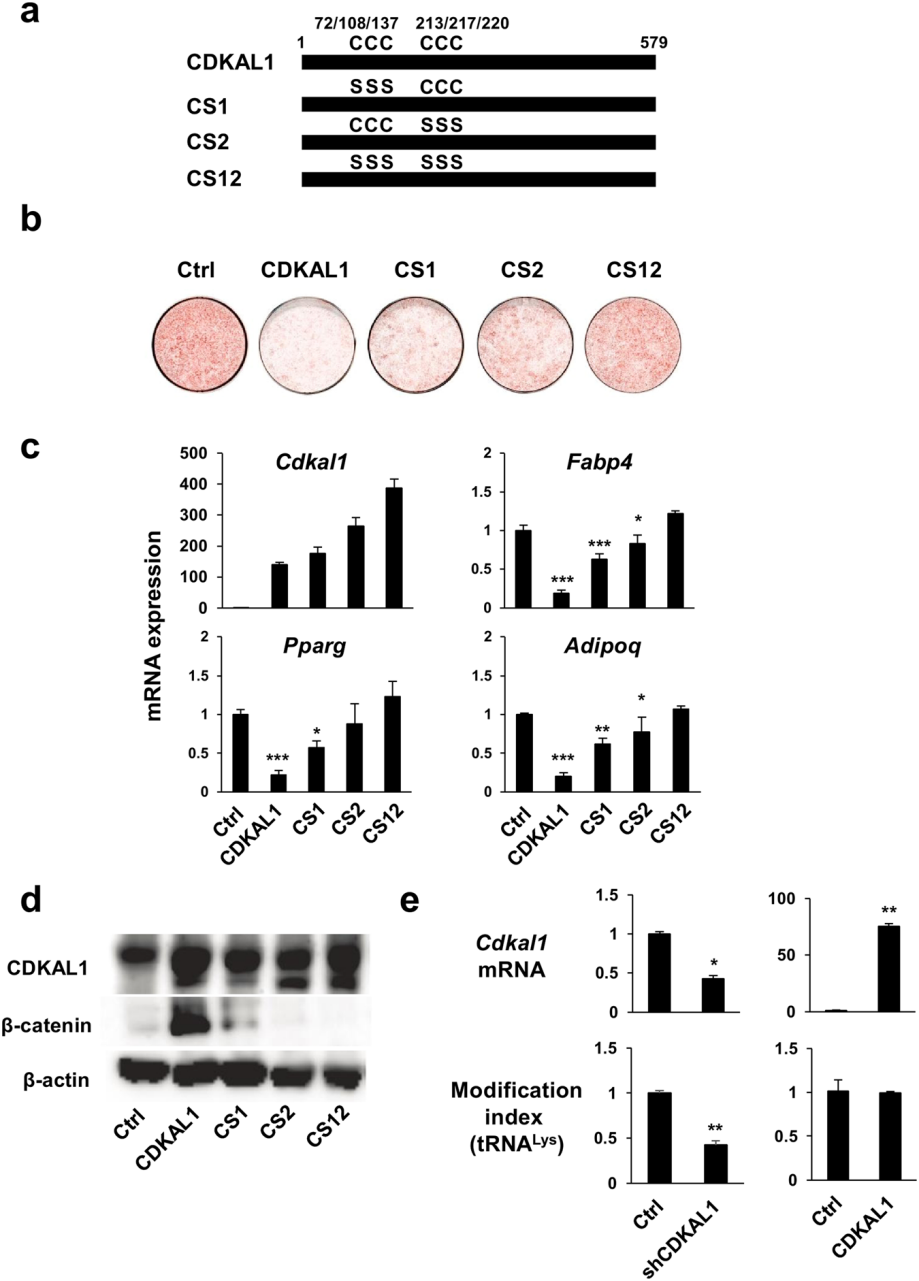
Conserved cysteine residues in the amino terminal region of CDKAL1 are essential for its anti-adipogenic action. (**a**) Diagram of constructs used in the experiment. The numbers indicate the amino acid residues from the N-terminus of CDKAL1. (**b**) Plate view of Oil Red O staining of differentiated 3T3-L1 cells expressing CDKAL1 with point mutations (Ctrl, control). (**c**) Gene expression levels in differentiated 3T3-L1 cells expressing CDKAL1 with the point mutations (n = 3, *p < 0.025, **p < 0.005, ***p < 0.0005 vs control group by Dunnett’s test). (**d**) Western-blotting analysis of β-catenin protein in differentiated 3T3-L1 adipocytes expressing CDKAL1 with the point mutations. (**e**) Measurement of tRNA^Lys^(UUU) modification index in 3T3-L1 cells expressing shCDKAL1 (*p < 0.05, **p < 0.01 vs control group by Student’s t-test) and in 3T3-L1 cells expressing wild type CDKAL1 (n = 3).

The amino terminal region of CDKAL1 contains six cysteine residues that form two clusters (4Fe-4S) conserved in the methylthiotransferase family^16^. We therefore made CDKAL1 with point mutations in which either three cysteine residues of the first Fe-S cluster (CS1) or those of the second cluster (CS2), or all six cysteine residues (CS12) were substituted for serine residues (Fig. 6a). The mutations at either CS1 or CS2 partially compromised the suppression of adipocyte differentiation by CDKAL1, and the mutations at both Fe-S clusters (CS12) largely compromised the anti-adipogenic action of CDKAL1 (Fig. 6b). Furthermore, 3T3-L1 cells expressing CDKAL1 CS mutants showed significant decrease in β-catenin levels and increased expression of the adipocyte-specific genes (Fig. 6c and d). The Fe-S clusters of CDKAL1 were previously shown to be critical for the methylthiotransferase activity of CDKAL1 that mediates synthesis of 2-methylthio-*N* ^*6*^-threonylcarbamoyladenosine in tRNA^Lys^(UUU)^16^. To test whether this action of CDKAL1 is involved the anti-adipogenic action of CDKAL1, we quantified the tRNA 2-methylthio modification of tRNA^Lys^(UUU) with a method using qPCR as reported by Xie P *et al*.^17^. Consistent with a previous report^11^, we observed significant reduction of the modification index when CDKAL1 was knocked down by shRNA in 3T3-L1 cells (Fig. 6e). However, we did not observe significant changes in the modification index in 3T3-L1 cells overexpressing CDKAL1 (Fig. 6e). These data imply that CDKAL1 suppresses adipocyte differentiation by an as yet unrecognized mechanism independent of its enzymatic activity on modification of tRNA^Lys^(UUU).

## Discussion

In our study, we investigated a role of CDKAL1 in 3T3-L1 adipocyte differentiation. Our present report demonstrates that CDKAL1 is expressed in adipocytes and that CDKAL1 negatively regulates adipocyte differentiation by activating the Wnt signaling pathway. Previous studies reported the influence of manipulation of the CDKAL1 gene expression on the body weight of mice. Okamura *et al*. reported that there was a small and transient reduction in the body weight and PPARγ expression in white adipose tissue of global CDKAL1 knockout mice during the early phase of high-fat feeding^13^. This seems to contradict what we observed in our study, but careful interpretation is required because the transient reduction of adipose tissue, accompanied by the reduction of food intake, is possibly caused by the action of CDKAL1 in the brain and also by the previously demonstrated actions of CDKAL1 in the pancreas^11, 12^. The Okamura group also reported that CDKAL1 transgenic mice under the control of the CAG promoter exhibited elevated adipose tissue mass and increased body weight^13^. This again seemingly contradicts our observation, but caution must be exercised because CDKAL1 is overexpressed by a ubiquitous CAG promoter. Indeed, the CDKAL1 transgenic mice under the control of the aP2 (*Fabp4*) promoter that we generated exhibited protection against obesity and insulin resistance under a high-fat-diet (Sun *et al*., unpublished data). Considering the fact that CDKAL1 is expressed in many tissues including adipose tissue, pancreatic islet, liver, muscle and the brain^11, 13^ and likely to have diverse functions, careful analyses and interpretation of the phenotype of the animals are required for elucidation of the precise roles of CDKAL1 in each tissue. The direct role of CDKAL1 in adipocyte differentiation that we demonstrated in this study provides a useful insight for interpretation of the complex phenotype. Nevertheless, we observed that CDKAL1 gene expression and protein levels were downregulated in *db*/*db* obese mice compared with wild-type C57BL/6 mice (Sun *et al*., unpublished data); clearly, it is important to investigate how CDKAL1 contributes to systemic glucose and energy homeostasis in physiological and pathological conditions.

The expression level of CDKAL1 is increased during adipocyte differentiation of 3T3-L1 and higher in the adipocyte fraction than in the stromal vascular fraction of adipose tissue (Fig. 1). Unexpectedly, the results of overexpression and knockdown experiments suggested that CDKAL1 has an anti-adipogenic action (Fig. 2). This makes a contrast with typical positive and negative regulators of adipocyte differentiation (e.g. PPARγ and C/EBPs for the former; Pref-1 for the latter), whose expression is increased or decreased, respectively, during differentiation. We speculate that CDKAL1 may function as a component that forms the negative feedback loop that prevents excess lipid accumulation in adipocytes. The activity of Wnt signaling is high and CDKAL1 is substantially expressed in the early stage of differentiation—expression level is one fourth of that of differentiated adipocytes (ref. 14 and Fig. 1). So we speculate that the activation of the Wnt pathway by CDKAL1 observed in the early stage of differentiation (Fig. 4a–c) is important for the anti-adipogenic action of CDKAL1. This notion is consistent with the fact that overexpression by electroporation of CDKAL1 in fully differentiated adipocytes was not sufficient to cause full suppression of the adipogenic genes (Suppl. Fig. 3).

The Wnt signaling pathway is one of the well-characterized negative regulators of adipocyte differentiation^18^. A study by the MacDougald laboratory first implicated Wnt signaling in the regulation of adipocyte differentiation^19^. Wnt signaling exerts its anti-adipogenic action through a number of mechanisms including both canonical and non-canonical Wnt signaling pathways, direct interaction and cross-regulation between β-catenin and PPARγ^18^. As shown in Fig. 4, CDKAL1 increases β-catenin and the active unphosphorylated form of β-catenin in the nucleus. In addition, we observed that CDKAL1 increased expression of Wnt target genes including *Wisp2* (Fig. 4g). Ulf *et al*. reported that WISP2 is an adipokine inhibiting adipogenesis. They demonstrated that secreted WISP2 directly activates the Wnt signaling pathway by promoting phosphorylation of Wnt co-receptors LRP5/6 and that cytosolic WISP2 inhibits PPARγ gene expression by retaining Zfp423^20^. These mechanisms are consistent with our finding that CDKAL1 overexpression inhibits PPARγ gene expression.

Our mutant studies suggest that the anti-adipogenic effect of CDKAL1 is dependent on two Fe-S clusters in the N-terminal region but presumably does not depend on the ability of CDKAL1 to modify the UUU anticodon in tRNA^Lys^(UUU)^11^ (Fig. 6). Especially given the fact that the anti-adipogenic action of CDKAL1 may be dependent on an enzymatic activity distinct from that on tRNA^Lys^(UUU), we speculate that various factors—including proteins in the Wnt pathway—could be the targets of CDKAL1. We specifically focused on PPARγ in this study, but other important regulators of adipocyte differentiation are also likely to be involved in the mechanism.

Genome-wide association studies showed that there was a significant association between SNPs in the CDKAL1 gene locus with either type 2 diabetes^1–4^, body mass index in adults and children^5–8^ or body weight at birth^9, 10^. Although we have demonstrated, here, that CDKAL1 is a negative regulator of adipocyte differentiation, we do not think that it is likely that the regulatory role of CDKAL1 in adipocyte differentiation can simply explain the effect of the CDKAL1 SNPs on body weight for these reasons: First, the risk SNPs of CDKAL1 is strongly associated with reduced insulin secretion rather than insulin resistance^1^. The SNPs with higher risk for type 2 diabetes are associated with lower body weight in adults and newborns^5, 7, 10^. These facts suggest that lower insulin secretion may mediate the indirect effect of the CDKAL1 SNPs on body weight. Second, the association between the risk SNPs and CDKAL1 gene expression levels is not clear^21, 22^. Nevertheless, a direct role of CDKAL1 rather than its SNPs on body weight cannot be excluded because CDKAL1 gene expression levels (but not the SNPs) in adipose tissue are inversely correlated with body mass index in human patients (unpublished data). Further effort to tease out the precise mechanisms by using improved genetic methods such as transancestral fine-mapping, high-density imputation^23^, by analyses on the association between the SNPs and tissue expression levels—expression quantitative trait loci^24^—and through epigenetic information such as long-range interaction^25, 26^—will be valuable.

In conclusion, the present study demonstrates that expression of CDKAL1 is induced during adipocyte differentiation. Functional assays showed that CDKAL1 is an inhibitor of adipocyte differentiation by activating the Wnt/β-catenin signaling pathway. Conserved cysteine residues of Fe-S clusters in the amino terminal region of CDKAL1 are essential for anti-adipogenic action. Our results imply that CDKAL1 serves as one of the negative regulators of adipocyte differentiation. Manipulation of CDKAL1 activity may be useful for treatment of obesity and type 2 diabetes.

## Methods

### Animals

Male C57BL/6 J mice were obtained from Charles River Laboratories, Inc. (Kanagawa, Japan). The animal care and use procedures were approved by the Animal Care Committee of the University of Tokyo. All experiments were performed in accordance with the University of Tokyo’s guidelines regarding animal research.

### Analysis of CDKAL1 mRNA expression in mouse tissues

Adipose tissues and pancreases were collected from five-month old C57BL/6 J mice. Each tissue was subjected to quantitative PCR measurement.

### Separation of primary adipocyte and stromal vascular fraction (SVF) from mice adipose tissues

Epididymal white adipose tissue was obtained from eight month old C57BL/6 J mice. Primary adipocytes and SVF were separated by collagen digestion. Each fraction was subjected to qPCR measurement.

### Luciferase reporter assays

3T3-L1 cells expressing CDKAL1 were plated in 24 well plates in DMEM (10% FBS) without antibiotics. The next day, cells were cotransfected with plasmids for either TOPFLASH (firefly luciferase gene under control of TCF/LEF response element, Millipore #17-285) or FOPFLASH (the negative control), LEF1, constitutively active mutant β-catenin (S37A) and renilla luciferase using Lipofectamine 2000 (Thermo Fisher Scientific K.K., Kanagawa, Japan). Twenty-four hours after transfection of plasmids, firefly and renilla luciferases were measured using the Dual-Luciferase Reporter Assay System (Promega KK, Tokyo, Japan) according to the manufacturer’s instructions. Firefly luciferase value was normalized to renilla luciferase.

### Plasmid construction

Murine CDKAL1 cDNA was cloned into pMXs-Puro retroviral expression vectors or pMXs-IRES-Blasticidin retroviral vectors (CELL BIOLABS, INC., CA, USA). CDKAL1 deletion mutants and CDKAL1 point mutants were constructed using the QuikChange Site-Directed Mutagenesis Kit (Agilent Technologies Japan, Ltd., Tokyo, Japan). The primer sequences used for point mutation are listed in Table S1. CDKAL1, β-catenin and luciferase shRNA constructs were designed using the siDesign-center (Thermo Fisher Scientific K.K). Sense and antisense oligos were cloned into pLMP plasmids (Thermo Fisher Scientific K.K.). The sense sequence for construction of shRNA is listed in Table S2.

### Cell culture

3T3-L1 cells were purchased from ATCC (VA, USA). 3T3-F442A cells were a gift from Dr. Peter Tontonoz of the University of California, Los Angeles, and the primary brown adipocyte cell line was from Dr. Shingo Kajimura of the University of California, San Francisco. Confluent 3T3-L1 cells and primary brown adipocytes were stimulated to differentiate with DMEM containing 10% FBS, 1 μM dexamethasone, 0.5 mM isobutylmethylxanthine, and 1 μM insulin (differentiation cocktail) for 2 days, followed by 1 μM insulin alone for 2 days. For differentiation of 3T3-F442A cells, only insulin was used. After the differentiation induction, cells were cultured with DMEM containing 10% FBS. When specified, PPARγ agonist pioglitazone was included.

### Retrovirus preparation

Plat-E cells were purchased from CELL BIOLABS, INC. (CA, USA) and cultured with DMEM containing 10% FBS, 1 µg/mL puromycin and 10 µg/mL blasticidin. Retroviruses were prepared by following the product manual. All experiments were approved by the Faculty’s committee of the University of Tokyo and were performed in accordance with the University of Tokyo’s guidelines regarding recombinant DNA.

### Retroviral infection

3T3-L1 preadipocytes were treated with conditioned medium from retrovirus producing Plat-E cells that were transfected with the retroviral vectors. Empty vectors (pMXs vectors or the pLMP vector) were used as controls. In some of the knockdown experiments, an shRNA construct targeting the luciferase gene was used as a negative control. After forty eight hours, 1 μg/mL puromycin or 10 μg/mL blasticidin was added to the culture medium and antibiotic-resistant cells were selected. The selected cells were then induced to differentiate to adipocytes by the method described above.

### Oil Red O staining

The 3T3-L1 adipocytes were washed with PBS and fixed with formalin for 30 minutes at room temperature. Fixed cells were rinsed with 60% isopropanol and stained with Oil Red O solution, which was freshly made by mixing 0.5% Oil Red O (Sigma-Aldrich) in isopropyl alcohol and water (3:2), then left to sit for 1 hour. The cells were then washed with water and dried.

### RNA extraction and quantitative PCR analysis

The total RNA from the mice tissues or cells were prepared by RNeasy kit (Qiagen Japan, Tokyo, Japan). RNA from each sample was converted to cDNA using the High Capacity RNA-to-cDNA Kit (Thermo Fisher Scientific K.K.). Real-time quantitative PCR (SYBR green) was performed on a 7900HT Fast Real-Time PCR System (Thermo Fisher Scientific K.K.). The primer sequences used are listed in Table S3. The other primers and probes were purchased from Applied Biosystems (Thermo Fisher Scientific K.K.). Gene expression levels were normalised to *Ppia* or *Rplp0*.

### Quantitative measurement of 2-methylthio modification of tRNA^Lys^(UUU)

The modification was analyzed according to the method reported by Xie P *et al*.^17^. Briefly, the reverse transcriptase reaction of DNase I-treated RNA samples was performed by using either reverse primer r1 or reverse primer r2 at 55 °C for 30 minutes. The cDNA samples were then subjected to quantitative PCR analysis by using a forward primer and the reverse primer r1. The primer sequences are listed in Table S4.

### Protein analysis

Protein preparation, SDS-PAGE, and western blotting were performed as previously described^27^. Anti-CDKAL1, anti-β-catenin and anti-adiponectin (19F1) antibodies were purchased from Abcam (Cambridge, UK) (ab169531, ab32572 and ab22554). Anti-β-actin and anti-PPARγ antibodies were purchased from Santa Cruz Biotechnology (TX, USA) (sc-1616R and sc-7196). Anti-Lamin A/C antibody, anti-GSK3-β and anti-phopho-GSK-3β were purchased from Cell Signaling Technology Japan, K.K. (Tokyo, Japan) (#2032 s, #9315 and #9336). Anti-non-phospho (active) β-catenin antibody was purchased from Merck Millipore (Darmstadt, Germany) (#05-665). Peroxidase-linked anti-rabbit or mouse IgG secondary antibody was purchased from ThermoFisher Scientific (Yokohama, Japan) (#65-6120 and A10668).

### Nuclear separation

Cells were collected by scraping into fresh PBS. After centrifugation at 1,850xG for 10 minutes, the supernatant was removed and the pellet was re-suspended in the fractionation buffer (250 mM sucrose, 20 mM HEPES pH7.4, 10 mM KCl, 1.5 mM MgCl_2_, 1 mM EDTA, 1 mM EGTA) and incubated on ice for 10 minutes. The cells were then forced to pass through a 25 G needle 10 times using a 1 ml syringe and incubated on ice for 20 minutes. The samples were centrifuged at 720xG for 5 minutes and the nuclei pellets were collected. The pellets were washed with the fractionation buffer again, centrifuged and re-suspended in the nuclear lysis buffer (1% NP40, 10 mM Tris-HCl, pH7.5, 150 mM NaCl, 10% glycerol, 0.1% SDS). The nuclear lysate was sonicated briefly on ice and centrifuged at 720xG for 10 minutes. The supernatant was used as the nuclear fraction.

### Statistical analysis

Data are expressed as mean ± standard deviation (*in vitro* studies) or standard error (*in vivo* studies). Statistical analyses were performed using Student’s t-test or ANOVA followed by one-tailed Dunnett’s test. P-values of <0.05 in the Student’s t-test or <0.025 in the one-tailed Dunnett’s test were considered significant.

## Electronic supplementary material

Supplementary Information
