# Supplementary material for: CDK5 Regulatory Subunit-Associated Protein 1-like 1 Negatively Regulates Adipocyte Differentiation through Activation of Wnt Signaling Pathway
Source: Sci Rep. 2017 Aug 4;7:7326. doi: 10.1038/s41598-017-06469-5 (PMC5544666; doi:10.1038/s41598-017-06469-5)
Supplement: Supplementary file 1 — Supplementary Information [file 41598_2017_6469_MOESM1_ESM.pdf]

CDK5 Regulatory Subunit-Associated Protein 1-like 1 Negatively Regulates Adipocyte Differentiation through Activation of Wnt Signaling Pathway

Kazumi Take[1], Hironori Waki[1,2,3*], Wei Sun[1], Takahito Wada[1], Jing Yu[1], Masahiro Nakamura[1], Tomohisa Aoyama[1], Toshimasa Yamauchi[1*] and Takashi Kadowaki[1*]

[1] Department of Diabetes and Metabolic Diseases, Graduate School of Medicine, The University of Tokyo, 7-3-1 Hongo, Bunkyo-ku, Tokyo 113-8655, Japan

[2] Functional Regulation of Adipocytes, Graduate School of Medicine, The University of Tokyo, 7-3-1 Hongo, Bunkyo-ku, Tokyo 113-8655, Japan

[3]Department of Molecular Sciences on Diabetes, Graduate School of Medicine, The University of Tokyo, 7-3-1 Hongo, Bunkyo-ku, Tokyo 113-8655, Japan

[*] Corresponding authors

Takashi Kadowaki (email: kadowaki-3im@h.u-tokyo.ac.jp), Toshimasa Yamauchi (email: tyamau-tky@umin.net), Hironori Waki (email: hwaki-tky@umin.ac.jp)

Supplementary Information

Table S1. Primers for mutagenesis

C72S	GATCCGAACATGGGGCAGCTCACATAATAATTCG
C108S	CTGTGGCTCCTGAACAGTAGTACTGTGAAAAATCCAG
C137S	TGTCCTCGCTGGCAGTGTTCCCCAAGC
C213S	TTTCCATCAACACGGGGAGTCTCAATGCTTGTACC
C217S, C220S	CACGGGGTGTCTCAATGCTAGTACCTACAGCAAAACTAAACAC

Table S2. Primer sense sequences used for the generation of shRNA constructs

CDKAL1	GCATGACAAATCCACCATATA
β-catenin	CAGATGGTGTCTGCCATTGTA
Luciferase	CCGCTGAATTGGAATCGATAT

Table S3. Primers for quantitative PCR

Adipoq	Fwd	CCGGAACCCCTGGCAG
	Rev	CTGAACGCTGAGCGATACACA
Adgre1	Taqman	Mm00802529_m1
Ccl2	Fwd	CATCCACGTGTTGGCTCA
	Rev	GATCATCTTGCTGGTGAATGAGT
Cdkal1	Fwd	CATCCATCAGCAAGCCACT
	Rev	TGATTCCCAAGCCTATTTCTG
Cebpa	Fwd	CCTTCAACGACGAGTTCCTG
	Rev	TGGCCTTCTCCTGCTGTC
Cebpb	Fwd	AAGAGCCGCGACAAGGC
	Rev	GTCAGCTCCAGCACCTTGTG
Cebpd	Fwd	TGCCCACCCTAGAGCTGTG
	Rev	CGCTTTGTGGTTGCTGTTGA
Cfd	Fwd	CTGGGAGCGGCTGTATGT
	Rev	CACGGAAGCCATGTAGGG
Cidec	Fwd	GATGGACTACGCCATGAAGTC
	Rev	GTGCTCACTGCCACATGC
Ctnnb	Fwd	GCAGCAGCAGTTTGTGGA
	Rev	TGTGGAGAGCTCCAGTACACC
Fabp4	Fwd	CACCGCAGACGACAGGAAG
	Rev	GCACCTGCACCAGGGC
Gja1	Fwd	TCCTTTGACTTCAGCCTCCA
	Rev	CCATGTCTGGGCACCTCT
Ppia	Taqman	Mm03024003_g1
Rarg	Fwd	TTTCCACCAGGTCCCTCAC
	Rev	CTGTCCAGTGGGTTTCCAAG
Rplp0	Fwd	AGATGCAGCAGATCCGCAT
	Rev	GTTCTTGCCCATCAGCACC
Pparg	Fwd	CCATTCTGGCCCACCAAC
	Rev	AATGCGAGTGGTCTTCCATCA
Wisp2	Fwd	TCCTCTGCATTCTCTCAATGG
	Rev	GTGTCCAAGGACAGGCACA

Table S4. Primers for quantitative measurement of 2-methylthio modification of $tRNA^{Lys}$(UUU)

Forward primer	GCATGACAAATCCACCATATA
Reverse primer 1 (r1)	CCTGGACCCTCAGATTAAAA
Reverse primer 2 (r2)	GAACAGGGACTTGAACCCTG

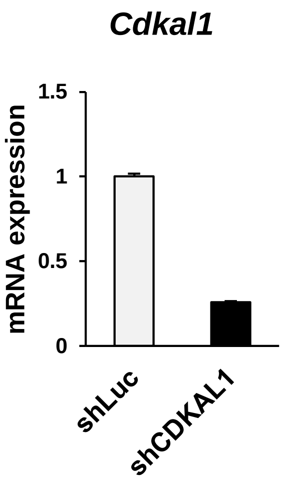

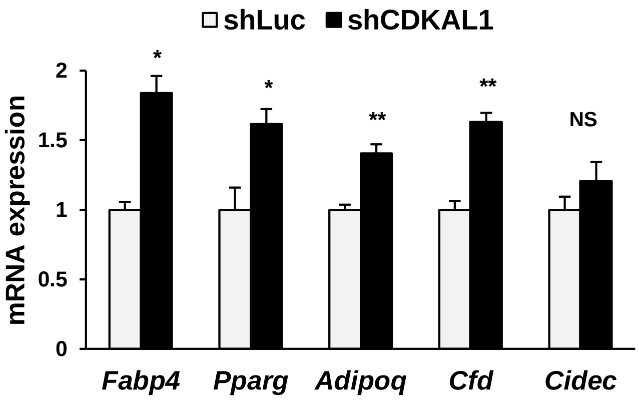

Suppl. Figure 1

The effect of stable knockdown of CDKAL1 by shRNA on the expression of the adipogenic genes in 3T3-L1 cells after differentiation. An shRNA for the luciferase gene was used as a negative control. (n = 3, * $p < 0.05$, ** $p < 0.01$ vs control group by Student's t-test)

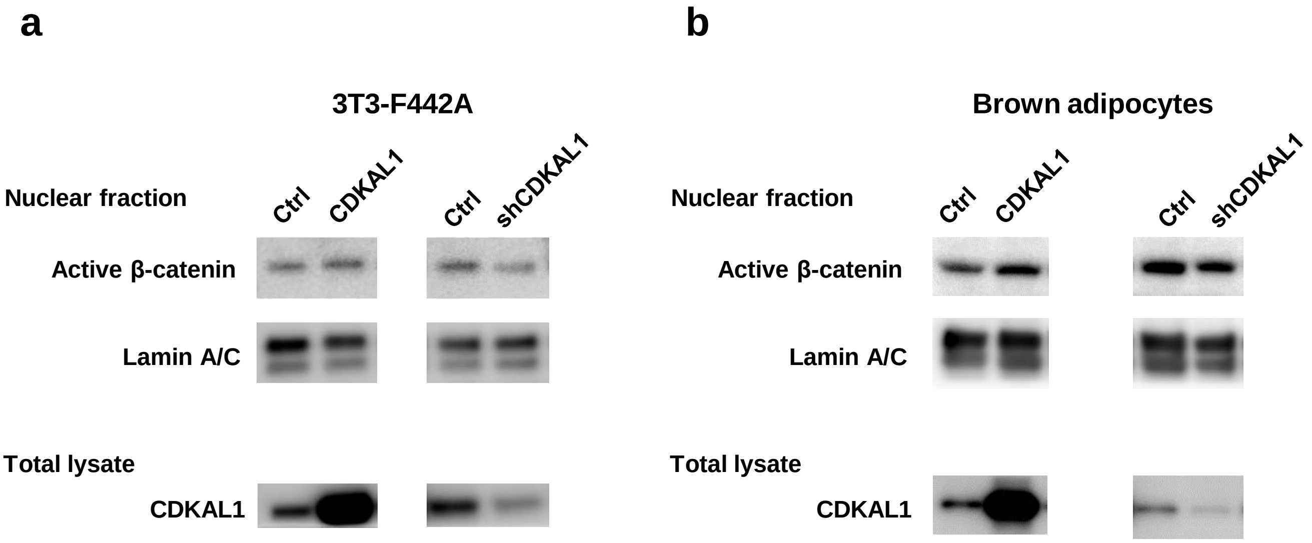

Suppl. Figure 2
The effect of either overexpression or knockdown of CDKAL1 on active β-catenin in the nuclear fraction in 3T3-F442A and brown adipocytes.

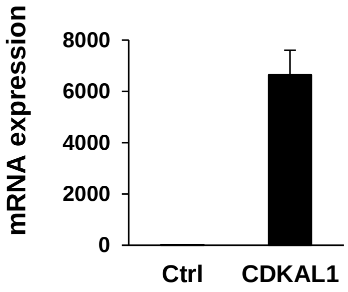

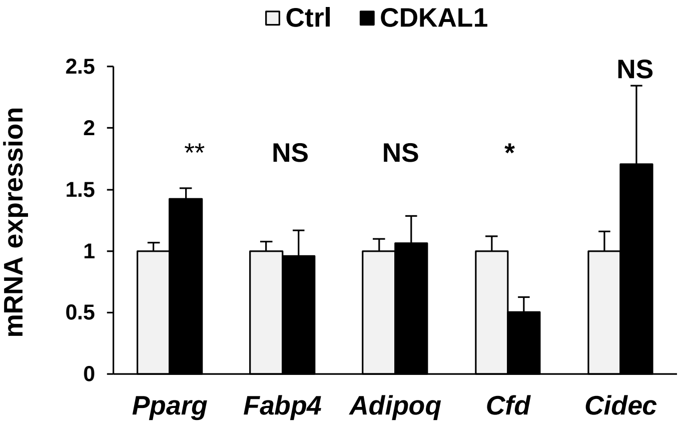

Suppl. Figure 3

Overexpression of CDKAL1 in fully differentiated adipocytes. Electroporation was performed on day 7 of differentiation and expression of indicated genes were measured 3 days after the electroporation. (n = 3, * $p < 0.05$, ** $p < 0.01$ vs control group by Student's t-test)
